# Predicting Adult Overweight and Obesity Prevalences Using the Food Nutritive Value Supplies of the FAO's Food Balance Sheet Data: Case Study of Trends in Spain

**DOI:** 10.1002/fsn3.71567

**Published:** 2026-02-22

**Authors:** Manuel Delgado‐Pertíñez, Sara Muñoz‐Vallés, José Luis Guzmán, Luis Ángel Zarazaga, Mao Chou Hsu, Michael López‐Herrera

**Affiliations:** ^1^ Departamento de Agronomía, Escuela Técnica Superior de Ingeniería Agronómica Universidad de Sevilla Sevilla Spain; ^2^ Departamento de Ciencias Agroforestales, Escuela Técnica Superior de Ingeniería, Universidad de Huelva, “Campus de Excelencia Internacional Agroalimentario, ceiA3” Campus Universitario de la Rábida Huelva Spain; ^3^ Department of Leisure and Sports Management Tajen University Yanpu Township Pingtung County Taiwan; ^4^ Escuela de Zootecnia, Centro de Investigación en Nutrición Animal Universidad de Costa Rica San José Costa Rica

**Keywords:** animal–vegetable foods, food balance sheet, multiple linear regression, obesity, overweight, Spain

## Abstract

Improving strategies against obesity and overweight requires estimating and constantly monitoring health risk factors. This study aimed to: (1) develop multiple linear regression (MLR) models predicting adult overweight and obesity prevalence in within‐country analysis, based on FAO food balance sheets, and (2) assess the evolution of food groups and the estimated adult overweight and obesity prevalences over recent decades in Spain as a case study, as well as analyzing their projections into the long‐term future by using three different FAO scenarios. The obtained MLR models showed high accuracy and predictive capability, and they would help to monitor these target health outcome variables in within‐country analysis. The evolution of whole consumption of food groups in Spain showed small or moderate variations from 2000 to the present. However, adherence to the traditional and healthy Mediterranean diet of the Spanish population decreased. Overweight and obesity prevalences in Spain, estimated by MLR, showed a downward trend or remained similar in the last decade. This was consistent with prevalence projections for 2050 in Spain according to the FAO Toward Sustainability scenario, predicting a decrease in the consumption of commodities supplying energy and protein per capita, and more aligned with current food‐based dietary guidelines in Spain. It will be necessary to assess whether this trend remains stable over time and whether it is the result of the changes in dietary patterns and lifestyles promoted by sectors involved in public health.

## Graphical abstract

**Objective:** This study aimed (1) develop multiple linear regression (MLR) models predicting adult overweight/obesity prevalences in within‐country analysis, based on FAO food balance sheets (FBSs), and (2) assess the evolution of estimated prevalences over recent decades in Spain as case study.

**Text from Table:** Final MLR models for prediction of adult overweight and obesity prevalences (E, energy; P, protein; F, fat).

**Text from Figures:** Spain's adult overweight/obesity prevalence before 2020 (a) and obesity prevalence (b) projections to 2050 under three FAO scenarios, modeled using MLR models and food supply data.

**Conclusion:** It will be necessary to assess whether that trend of overweight/obesity prevalences in Spain remains stable over time and whether it is the result of the changes in dietary patterns and lifestyles promoted by sectors involved in public health.

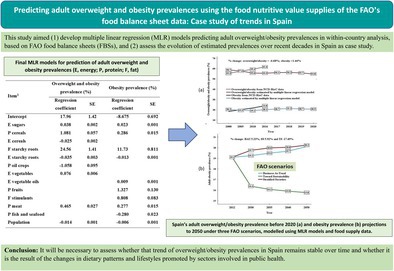

## 1 Introduction

Overweight (body mass index [BMI] 25.0–29.9 kg/m^2^) and obesity (BMI ≥ 30.0 kg/m^2^; cutoff criteria based on the World Health Organization [WHO] standards; WHO Expert Consultation 2004) are associated with high health costs and an increased risk of mortality (Cawley and Meyerhoefer 2012; Cerrillo et al. 2023). These conditions are considered major risk factors for a range of chronic diseases, such as cardiovascular disease, type 2 diabetes mellitus, musculoskeletal diseases, and some cancers (WHO 2009; Ng et al. 2014; The GBD 2017). The widespread rise in obesity, in both high‐income and low‐ and middle‐income countries, is one of the most complex and costly public health challenges facing society today (Aranceta‐Bartrina et al. 2016; WHO 2022). In Europe and the USA, for instance, the prevalence of obesity has dramatically increased over the past four decades (Janssen et al. 2020). The WHO developed the Global Action Plan for Prevention and Control of Noncommunicable Diseases 2013–2020 to stop the increase in obesity between 2010 and 2025 (WHO 2013).

It is well documented that diet and lifestyle are the main factors affecting overweight and obesity rates (You and Henneberg 2016). Energy intake is considered a major factor related to the increase in these rates. However, increasing evidence suggests that some dietary patterns have a higher contribution to promoting overweight than others (Mozaffarian et al. 2011). Nevertheless, little knowledge exists of how diets containing different compositions of food groups or macronutrients may be determinants for the development of obesity, particularly in terms of population‐level assessment (You and Henneberg 2016). Evaluating and monitoring food consumption and diet quality is thus essential because this would help identify those essential dietary patterns to improve the population's nutritional status (Varela‐Moreiras et al. 2010; Cerrillo et al. 2023).

Food balance sheets (FBSs), developed by the Food and Agricultural Organization (FAO), measure the food supply of the population (food available per capita) and provide an approximate picture of the overall food situation in a country (FAO 2001). Although FBSs can mainly overestimate results, and reliability depends on the basic input data, they have been applied in many health investigations from within‐country food and nutrition monitoring to international studies, as well as modeling analyzes (Thar et al. 2020). The FAO report “Food and Agriculture Projections to 2050” (FAO 2018a) introduced per capita energy (kcal) and protein (g) supply projections based on three defined trajectories that represent alternative future food systems, comprising a Business as Usual (BAU) scenario, assuming the continuation of historical trends in food preferences; a Stratified Societies (SS) scenario, emphasizing the consequences of ignoring the current and future challenges in food and agricultural systems and leaving them unattended; and a Toward Sustainability (TS) scenario, visualizing a more equitable global society whereby several Sustainable Development Goal (SDG) targets are nearly universally achieved and food systems move toward sustainability through the adoption of effective policies.

Overweight and obesity in Spain currently show high prevalence. According to the latest Spanish Health Survey (ENSA) from the Spanish Ministry of Health (MSCB 2017), although this type of data tends to underestimate the prevalences (Nyholm et al. 2007; Acevedo et al. 2014) since people tend to underestimate their weight and overestimate their height, more than half (54.5%) of adults are overweight in Spain. Additionally, the survey highlights an upward trend, indicating that, in the last 30 years, the prevalence of obesity in adults has multiplied by 2.4, from 7.4% in 1987 to 17.4% in 2017. Earlier studies based on anthropometric data, such as the ENRICA study (between 2008 and 2010; Gutiérrez‐Fisac et al. 2012), the ANIBES study (in 2013; López‐Sobaler et al. 2016), and the ENPE study (between 2014 and 2015; Aranceta‐Bartrina et al. 2016), also reported rising trends among adults. However, some data, such as those from the European Health Surveys in Spain (EHSS) from 2009 and 2014 (Acevedo et al. 2017), despite the limitation of analyzing self‐reported data, have confirmed a trend toward lower overweight and obesity prevalence levels in the last decade. These results would be more aligned with the current food‐based dietary guidelines (more sustainable and healthy diet) of Spain (IPCC 2019; Aranceta‐Bartrina et al. 2019). These statistics necessitate constantly estimating and monitoring obesity and overweight levels in the Spanish population to improve strategies against these health risk factors.

The primary aim of the present work was to develop multiple linear regression (MLR) models using FAO FBS data (per capita food supplies, expressed in terms of caloric value and protein and fat content) to predict the prevalence of health outcome variables (adult overweight and obesity); FBSs are updated regularly and standardized for all countries so the models obtained would help to monitor these variables and examine the effects of policies and interventions. A second aim of the current work was to examine the evolution of animal–vegetable sourced foods and the estimated adult overweight and obesity prevalences in Spain, as a case study, from 2000 to 2020, as well as to analyze their projections into the long‐term future using three different FAO scenarios.

## 2 Methods

### 2.1 Data Sources and Compilation

This study was based on data collected from the FAO Corporate Statistical Database (FAOSTAT), covering the period from 2000 to 2020. Regarding adult obesity and overweight prevalences, data were collected from the FAO dataset containing the Suite of Food Security Indicators (Available between 2000 and 2016), originally sourced from the WHO Global Health Observatory. Since nutrition data were not age‐ or sex‐specific, the results were reported for both sexes together.

Due to the lack of a long‐term national dietary intake dataset, and to avoid inter‐country variations and potential errors, data about food and nutrient availability were obtained from the FAO's FBSs, which are compiled and documented in the FAOSTAT database. The previous FBS data from 2000 to 2013 (FAO 2022a) and updated FBS from 2014 to 2020 (FAO 2022b) were considered and used during our data extraction period.

The FBSs show the sources of supply and utilization for each food item. They are compiled by subtracting utilization (quantity exported, fed to livestock, used for seed, manufactured for food and non‐food uses, and storage‐ and transportation‐related losses) from the total supply (quantity imported and produced, with adjustments for changes in stocks). The per capita supply of each such food item available for human consumption is then calculated by dividing the corresponding amount by the population size of a given country (FAO 2001, 2022a, 2022b). Data about per capita food supplies are expressed in terms of quantity and, by applying appropriate food composition factors for all primary and processed products, also in terms of caloric value and protein and fat content (g).

Overweight and obesity were assessed by considering BMI as an indicator of relative weight and obesity. Prevalences of overweight and obesity in adults were based on the WHO cutoff criteria (WHO 2004), which consider the population with a BMI of 25 kg/m^2^ or higher to be overweight and the population with a BMI of 30 kg/m^2^ or higher to have obesity. Prevalences were reported as the proportion of the population over 18 years of age, standardized by age and weighted by sex (WHO 2022). The WHO Global Health Observatory data repository is the present source of FAOSTAT data, which, in turn, uses data obtained from the NCD Risk Factor Collaboration (NCD‐RisC) study (NCD‐RisC 2017). These data comprise the available measured height and weight information, supplemented with estimates from a Bayesian hierarchical model based on measured height and weight data combined with data from other years and countries, to estimate trends in mean BMI and prevalence of BMI categories (underweight, overweight, and obesity) from 1975 to 2016.

Future projections were analyzed with data obtained from the FAOSTAT portal, in which FAO has projected the national energy and protein demand per capita to 2050 (FAO 2018b). The same method was adopted as previously outlined (per capita per day), where projected energy (kcal) and protein (g) supply data were downloaded from the FAO database (FAO 2018b). The projections were based on three defined trajectories representing alternative future food systems, as explained in the FAO report “Food and Agriculture Projections to 2050” (FAO 2018a) and recently described by Henchion et al. (2021). The first scenario, BAU, assumes a continuation of historical trends in food preferences. In this projection, efforts are made to meet and maintain SDG targets, but these ultimately fail to address many of the issues facing food and agriculture. The second scenario, SS, emphasizes the consequences of ignoring the current and future challenges in food and agricultural systems. The third scenario, TS, forecasts a more equitable global society whereby several SDG targets are almost universally achieved and food systems move toward sustainability through the adoption of effective policies. All scenarios use the same population projections. Regarding dietary evolution, the overview of assumptions in each FAO scenario is as follows: BAU, current trends continue with a moderate convergence toward the consumption of more nutritious food; SS, diets worsen for most people due to lower purchasing power and lessened consumer awareness while elites consume high‐quality luxury foods; TS, balanced, healthy, and environmentally sustainable diets are mostly universally adopted.

### 2.2 Statistical Analysis

MLR models were developed to predict adult overweight and obesity, as well as obesity prevalences in each country (dependent variables) from the annual changes in the population and daily per capita food supplies, using FAO FBS data (expressed in terms of caloric value and protein and fat content; explanatory variables). The dataset was restricted to 2000–2016, the most recent records available for the health variables, in accordance with Janssen et al. (2020). These authors obtained an obesity forecast by linearly extrapolating the rate of change of the logistically transformed obesity prevalence into the future. They also extrapolated the transformed period from 2000 onwards because the declining rate of change was more stable during this period compared to earlier previous years. The commodity groups included in the study were cereals (excluding beer), fruits, tree nuts, vegetables, vegetable oils, sugars and sweeteners, starchy roots, pulses, spices, oil crops, stimulants, alcoholic beverages, meat, eggs, milk (excluding butter), fish and seafood, and animal fats and remnants. Countries with missing data and unstable borders over time were not taken into consideration, resulting in a final dataset of 163 countries.

Before the analysis, the initial dataset was randomly divided into two different subsets: a model calibration dataset (70% of the entire dataset) and a validation dataset (the remaining 30%). The calibration dataset was used to generate the prediction models, which were applied to the validation dataset to quantify their predictive ability. The prediction model variables were selected using an automatic stepwise selection with slstay = 0.05 and slentry = 0.15. Potential multicollinearity among independent variables was verified by preliminary correlation analyzes and the variance inflation factor (VIF). With a VIF of ≥ 10, collinearity was suspected; the variable was removed, and the models were rebuilt. This process was implemented until the VIF for all variables was < 10.

The normality of the model predictions was examined by exploring residual plots from studentized residuals. The optimal number of terms was chosen as the minimum number of terms that obtained a low standard error of prediction and kept the adjusted *R*‐square value practically the same. The goodness‐of‐fit statistics considered were the coefficient of determination from the calibration and validation (*R* ^2^_C_ and *R* ^2^_V_, respectively) datasets and standard error of prediction from the calibration and validation (SEP_C_ and SEP_V_, respectively) datasets. To evaluate the practical utility of the prediction models, the ratio of performance to deviation (RPD) and concordance correlation coefficient (CCC) were estimated (Visentin et al. 2015). The RPD was determined as the ratio of the standard deviation of the variable and the standard error of prediction from the prediction model (Williams 2007). The CCC was determined as (Lawrence and Lin 1989):

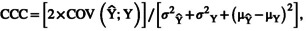

where COV (Ŷ; Y) is the covariance between the reference (Y) and predicted values (Ŷ), *σ* ^2^_Y_ is the variance of the reference values, *σ* ^2^_Ŷ_ is the variance of the predicted values, and μ_Y_ and μ_Ŷ_ represent the mean of the reference and predicted values, respectively. The bias for each prediction model was determined as the average of the difference between the reference value and the respective predicted value; a *t*‐test was used to determine whether this was significantly different from zero.

For the projections of adult overweight and obesity prevalences into the long‐term future, using the same FAO FBS data and considering three different FAO scenarios, another MLR analysis was performed using the same FAO FBS data, using only food groups that FAO considers for projections and expressed only in terms of caloric value and protein content. To this aim, the food groups of cereals, fruits, oil crops, starchy roots, pulses, vegetables, vegetable oils, sugars and sweeteners, stimulants, meat, raw milk, and fish were extracted from the dataset. No food group was dedicated to eggs, animal fats, offal, alcoholic beverages, tree nuts, or spices. The same method previously outlined was adopted to build the MLR models. The final models obtained were used to estimate adult overweight and obesity prevalences toward 2050 (Table S1, Figure S1).

All statistical analyzes were performed with IBM SPSS Statistics for Windows (version 26.0; IBM Corp., Armonk, NY, USA).

## 3 Results

### 3.1 Multiple Linear Regression Models

**FIGURE 1:**
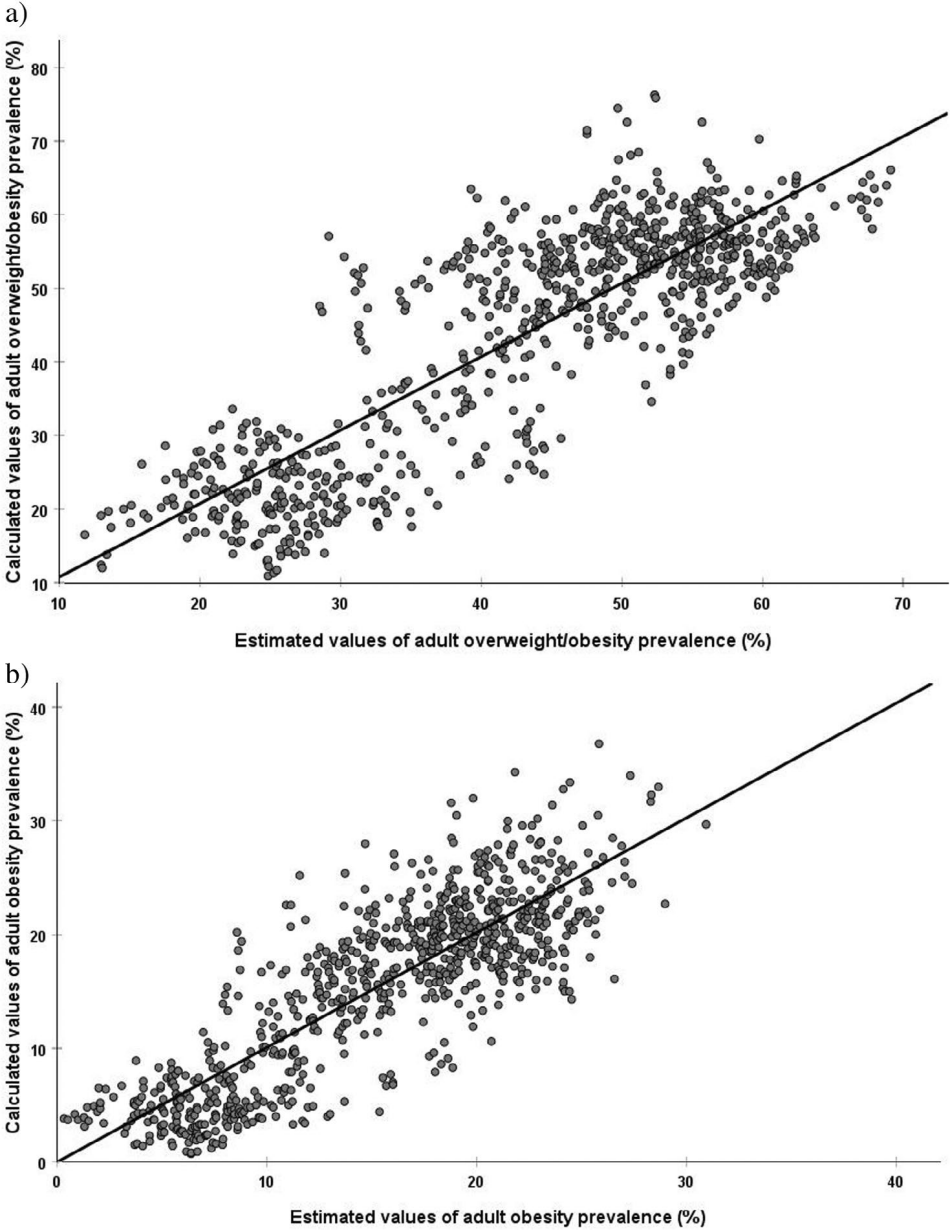
Graphic representation of calculated values of (a) adult overweight and obesity (BMI ≥ 25 kg/m^2^) and (b) obesity (BMI ≥ 30 kg/m^2^) prevalences and their estimation from multiple linear regression with split‐sample validation (30%), applying the developed prediction model from Table 1.

**FIGURE 2:**
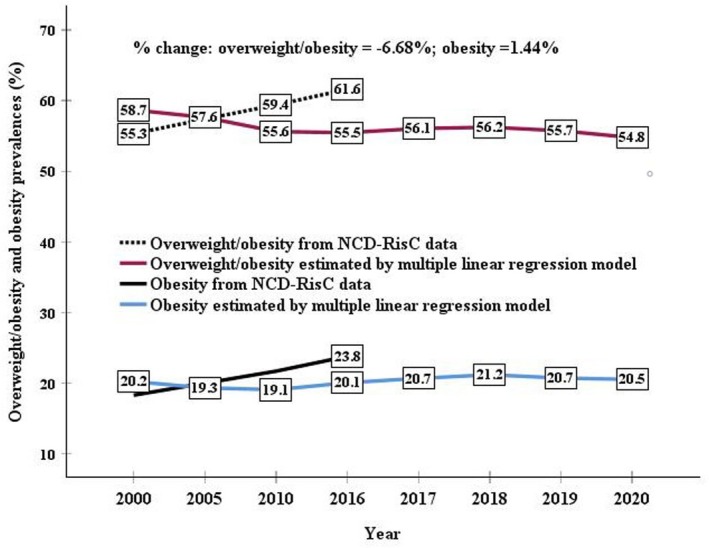
Overweight and obesity (BMI ≥ 25 kg/m^2^) and obesity (BMI ≥ 30 kg/m^2^) prevalences in the adult Spanish population according to NCD‐RisC 2017 data (from 2000 to 2016) or estimated by multiple linear regression model (from 2000 to 2020) based on food nutritive value supplies obtained from FAO food balance sheets. The percentage change from 2000 to 2020 using estimated data was also calculated, based on the calculation: 100 × [(2020 value−2000 value)/2000 value].

The final MLR models and retained prediction variables for adult overweight and obesity prevalences are presented in Table 1 and Figures 1 and 2. The goodness‐of‐fit statistics of the prediction models are summarized in Table 2. The MLR models used for the projections of adult overweight and obesity prevalences are presented in the supplementary results (Table S1, Figure S1). The variables retained in the predictive models for overweight and obesity prevalence included the nutritional contributions of sugars (energy), cereals (energy and protein), starchy roots (fat and energy), oil crops (protein), vegetables (energy), and meat (protein). The nutritional contributions of sugars (energy), cereals (protein), starchy roots (fat and energy), vegetable oils (energy), fruits (protein), stimulants (fat), meat (protein), and fish and seafood (protein) were retained as variables to predict obesity prevalence (Table 1).

**TABLE 1:** Final multiple linear regression models for prediction of adult overweight and obesity (BMI ≥ 25 kg/m^2^) and adult obesity (BMI ≥ 30 kg/m^2^) prevalences based on food nutritive value supplies obtained from FAO food balance sheets (per capita and day).

Item^a^	Adult overweight and obesity prevalence (%)			Adult obesity prevalence (%)		
	Regression coefficient	SE	*p*	Regression coefficient	SE	*p*
Intercept	17.96	1.42	< 0.001	−8.675	0.692	< 0.001
E sugars	0.038	0.002	< 0.001	0.023	0.001	< 0.001
P cereals	1.081	0.057	< 0.001	0.286	0.015	< 0.001
E cereals	−0.025	0.002	< 0.001			
F starchy roots	24.56	1.41	< 0.001	11.73	0.811	< 0.001
E starchy roots	−0.035	0.003	< 0.001	−0.013	0.001	< 0.001
P oil crops	−1.058	0.095	< 0.001			
E vegetables	0.076	0.006	< 0.001			
E vegetable oils				0.009	0.001	< 0.001
P fruits				1.327	0.130	< 0.001
F stimulants				0.808	0.083	< 0.001
P meat	0.465	0.027	< 0.001	0.277	0.015	< 0.001
P fish and seafood				−0.280	0.023	< 0.001
Population, million	−0.014	0.001	< 0.001	−0.006	0.001	< 0.001

^a^ E, energy (kcal); P, protein (g); F, fat (g).

**TABLE 2:** Fitting statistics^a^ in calibration model and validation for prediction of adult overweight and obesity (BMI ≥ 25 kg/m^2^) and adult obesity (BMI ≥ 30 kg/m^2^) prevalences based on food nutritive value supplies obtained from FAO food balance sheets (per capita and day).

Item	*n*	*R* ^2^	SEP	RPD	CCC	Bias
**Adult overweight and obesity prevalence (%)**						
Calibration model	1746	0.78	7.44	2.14	0.87	−0.29
Validation	830	0.72	8.21	1.94	0.85	−0.50
Adult obesity prevalence (%)						
Calibration model	1746	0.73	4.28	1.99	0.84	−0.08
Validation	829	0.71	4.21	2.03	0.83	−0.11

^a^ R^2^, coefficient of determination; SEP_,_ standard error of prediction; RPD, ratio performance deviation; CCC, concordance correlation coefficient; Bias, average difference between the reference value and the respective predicted value.

The accuracy of predicting adult overweight and obesity prevalences (Table 2) was high, with a respective R^2^_C_ of 0.78 and 0.73 and a SEP_C_ of 7.44 and 4.28. Additionally, the models exhibited a high predictive ability. The calibration dataset RPD values ranged from 2.14 (overweight and obesity) to 1.99 (obesity), whereas the CCC was between 0.87 (overweight and obesity) and 0.84 (obesity). Similar results were obtained to the validation dataset, where RPD values were between 1.94 (overweight and obesity) and 2.03 (obesity), and the CCC ranged from 0.85 (overweight and obesity) to 0.83 (obesity). The mean bias of prediction in the estimation of the prevalence of overweight and obesity showed no difference (*p* > 0.05) from zero.

### 3.2 Trends in Animal–Vegetable Food Consumption in Spain and Long‐Term Future Projections

Milk, cereals, vegetables, fruits, meat, and alcoholic beverages constituted the main food groups (≥ 100 kg/capita/year) for Spanish consumption over the last two decades (Table 3). Except for cereals, whose consumption has increased moderately over time (about 15%), the consumption of each main food slightly decreased (less than 10%), except vegetables, which showed the strongest downward trend over time (35%), from 165 kg/year to 107 kg/year between 2000 and 2020. Other relevant food groups were starchy roots (about 60 kg/capita/year), fish and seafood (about 40 kg/capita/year), and vegetable oils and sugars and sweeteners (with a consumption of each of these close to 30 kg/capita/year). The consumption patterns over time for these last foods were not homogenous, with slight increases for vegetable oils and sugars and sweeteners, slight decreases for fish and seafood, and a modest decrease for starchy roots. Finally, the lowest consumed groups of foods (< 10 kg/capita/year) were eggs, tree nuts, pulses, oil crops, stimulants, animal fats, offal, and spices. Trends for these foods over time showed heterogeneity, nonetheless. Except for animal fats showing a slight decrease, offal, oil crops, and stimulants showed a sharp decrease (between 35% and 45%). In contrast, the pattern of egg and pulse consumption was more stable over time (with only slight increases), whereas tree nuts and spices showed a pronounced increasing trend (83% and 50%, respectively).

**TABLE 3:** Evolution of food supply for major commodities in Spain from 2000 to 2020. The percentage change from 2000 to 2020 is also presented.

Food commodities	Per capita food supply (kg/year)^a^					% change^b^
	2000	2005	2010	2015	2020	
Cereals (excluding beer)	99.0	95.9	103.0	114.0 (313)	114.4 (313)	15.5
Fruits	107.4	100.2	79.7	64.8 (178)	98.3 (269)	−8.5
Tree nuts	7.1	7.8	6.8	5.3 (15)	13.0 (36)	83.1
Vegetables	164.6	155.8	140.5	125.5 (344)	106.5 (292)	−35.3
Vegetable oils	28.2	26.0	27.0	28.2 (77)	30.8 (84)	9.2
Sugars and sweeteners	29.6	29.9	30.4	33.3 (91)	31.2 (85)	5.4
Starchy roots	76.6	68.7	64.5	57.0 (156)	58.4 (160)	−23.8
Pulses	5.6	4.6	5.2	5.0 (14)	5.9 (16)	5.4
Spices	0.2	0.4	0.3	0.2 (0.5)	0.3 (0.8)	50.0
Oil crops	7.8	5.4	5.6	3.0 (8)	5.2 (14)	−33.3
Stimulants	6.6	7.2	6.9	3.9 (11)	3.6 (10)	−45.4
Alcoholic beverages	108.4	113.9	100.6	98.8 (271)	105.6 (289)	−2.6
Meat	113.2	108.1	96.6	97.6 (267)	101.9 (279)	−10.0
Eggs	13.9	12.9	13.7	13.8 (38)	14.8 (41)	6.5
Milk (excluding butter)	168.0	159.5	172.3	151.3 (415)	156.6 (429)	−6.8
Fish and seafood	42.3	41.4	43.0	43.6 (119)	40.8 (112)	−3.5
Animal fats	4.8	4.9	5.2	4.9 (13)	4.6 (13)	−4.2
Offal	5.8	5.4	5.4	4.4 (12)	3.8 (10)	−34.5

^a^ Between parenthesis g/day.

^b^ Based on the calculation 100 × (2020 value−2000 value)/2000 value.

In terms of the contribution of animal‐ and vegetable‐sourced foods to the energy, protein, and fat supply between 2000 and 2020 in Spain, according to the FAO FBSs (Table 4), animal protein was more relevant than vegetable protein (around 64% of the total supplied protein), in contrast to energy (around 27% of total energy) and fat (around 40% of total fats). The overall contribution of different macronutrients to the diet over time was generally stable, although major changes were detected depending on the origin of the food source, particularly concerning energy and fats. The contribution of animal‐sourced foods to total consumption showed a decreasing trend in both parameters (approximately 6% and 11.5% for total energy and fat, respectively). Concerning the calculated percentage contributions of proteins, fats, and carbohydrates to the total energy supply, in general, energy from carbohydrates was around 45%, fats provided around 42% of energy, and proteins provided approximately 13% of the supplied energy (Table 4). Concerning carbohydrates and fat, trends over time remained relatively constant throughout the study period, whereas energy from protein fell slightly (1.5%).

**TABLE 4:** Food supply in dietary energy (DES) and macronutrients in Spain from 2000 to 2020. The contributions to total DES and the percentage change from 2000 to 2020 are also presented.

Parameter^a^	Per capita/day supply					% change^b^
	2000	2005	2010	2015	2020	
E total	3360	3220	3183	3195	3354	−0.2
E from animal products	949	902	852	871	893	−5.9
E from vegetable products	2411	2318	2330	2324	2461	2.1
P total	112.2	108.7	107.0	105.4	111.1	−1.0
P from animal products	71.7	69.8	67.4	66.5	71.0	−1.0
P from vegetable products	40.5	38.9	39.5	38.8	40.2	−0.7
F total	155.1	145.9	144.0	147.2	155.1	0.0
F from animal products	64.4	60.9	56.6	58.9	57.0	−11.5
F from vegetable products	90.7	85.0	87.4	88.3	98.0	8.0
Contribution of fats to total DES (%)	41.5	40.8	40.7	41.5	41.6	0.2
Contribution of carbohydrates to total DES (%)	45.1	45.7	45.9	45.3	45.2	0.2
Contribution of protein to total DES (%)	13.4	13.5	13.4	13.2	13.2	−1.5

^a^ E, energy, in kcal; P, protein, in g; F, fats, in g.

^b^ Based on the calculation 100 × (2020 value−2000 value)/2000 value.

Regarding projections of food supply in dietary energy and protein (capita/day) of major food commodities in Spain to 2050, each of the three FAO scenarios had a different result for the future food supply, depending on the conjectures considered (Table 5). From a whole perspective, a slight increase (≤ 10%) in total energy and protein demand was predicted in 2050 across BAU and SS scenarios when compared to the BAU 2012 baseline, whereas a slight decrease was predicted for the TS scenario. In general, slight increases (3%–6%) and a notable decrease (20%–35%) were observed in the demand for milk and fish macronutrients, respectively, across all projections and in each of the analyzed years. For the rest of the food commodities, slight increases were observed in the demand for energy and protein in the BAU and SS scenarios in each year analyzed, except for meat, which showed a larger increase, especially in the SS scenario (around 20%). In contrast, the TS scenario presented slight and moderate proportional declines in food supply macronutrients when compared to the 2012 BAU baseline.

**TABLE 5:** Projections of food supply in dietary energy and protein (capita/day) of major food commodities in Spain to 2050, based on three different FAO scenarios. Also presented is the forecasted percentage change in food supply comparing 2012 BAU with each of the 2050 scenario projections.

Food supply^a^	2012	2030			2040			2050			2012 BAU vs. 2050		
	BAU/SS/TS	BAU	SS	TS	BAU	SS	TS	BAU	SS	TS	BAU	SS	TS
E total	3185	3249	3237	3044	3261	3272	2982	3265	3311	2929	2.5	4.0	−8.0
E cereals	793.3	832.7	822.4	791.1	837.6	841.2	779.6	840.3	861.9	766.1	5.9	8.6	−3.4
E vegetable oils	664.3	673.5	669.8	640.3	673.5	677.4	628.2	672.4	688.1	616.1	1.2	3.6	−7.3
E sugars	290.0	305.8	278.2	261.8	309.3	278.4	250.3	311.6	282.0	242.3	7.5	−2.8	−16.4
E starchy roots	108.7	109.6	109.8	108.2	110.0	110.4	107.6	110.3	110.8	106.9	1.5	2.0	−1.6
E fruits	88.0	88.6	88.1	83.4	88.9	88.9	80.6	88.8	89.1	77.6	0.9	1.3	−11.8
E vegetables	85.0	88.3	87.3	82.7	89.4	89.0	80.9	90.6	90.4	79.4	6.5	6.4	−6.6
E meat	369.3	388.6	431.8	326.5	398.4	438.1	316.8	409.5	438.5	315.3	10.9	18.7	−14.6
E milk	272.7	280.2	286.5	270.7	281.3	287.9	274.2	281.8	288.8	280.8	3.4	5.9	3.0
E fish	84.3	72.1	65.2	70.0	69.7	65.4	64.1	65.6	66.6	56.1	−22.2	−21.1	−33.5
P total^b^	96.6	98.2	100.7	90.2	98.9	102.0	88.0	99.3	103.0	86.3	2.8	6.6	−10.6
P cereals	24.4	25.5	25.2	24.2	25.6	25.8	23.8	25.7	26.5	23.4	5.8	8.6	−3.9
P meat	30.8	32.5	36.1	27.1	33.3	36.6	26.5	34.2	36.5	26.4	11.1	18.8	−14.3
P milk	14.7	15.1	15.5	14.6	15.2	15.5	14.8	15.2	15.6	15.1	3.4	5.9	3.0
P fish	12.5	10.7	9.0	10.4	10.3	9.7	9.5	9.7	9.9	8.3	−22.2	−21.1	−33.5

Abbreviations: BAU = Business as Usual, SS = Stratified Societies, TS = Toward Sustainability.

^a^ Per capita/day; E, energy in kcal; P, protein in g.

^b^ Excluding the following foods: eggs, animal fats, offal, alcoholic beverages, tree nuts, and spices.

### 3.3 Trends of Overweight and Obesity in Spain and Long‐Term Future Projections

Figure 2 shows the overweight and obesity prevalences in the adult Spanish population, according to the NCD‐RisC (2017) data (from 2000 to 2016) or estimated by the MLR model (from 2000 to 2020), based on food nutritive value supplies obtained from the FAO's FBSs. Although both types of data showed similar values, NCD‐RisC data showed an upward trend in overweight and obesity rates over time. For instance, in the last year of the NCD‐RisC data (2016), overweight and obesity prevalences were 61.6% and 55.5%, respectively, and obesity prevalences were 23.8% and 20.1%, according to NCD‐RisC data or estimated by MLR, respectively. In contrast, the overweight and obesity prevalences estimated by MLR decreased over time (6.7% change from 2000 to 2020), whereas the obesity rate remained stable or experienced slight increases (1.4% change from 2000 to 2020).

**FIGURE 3:**
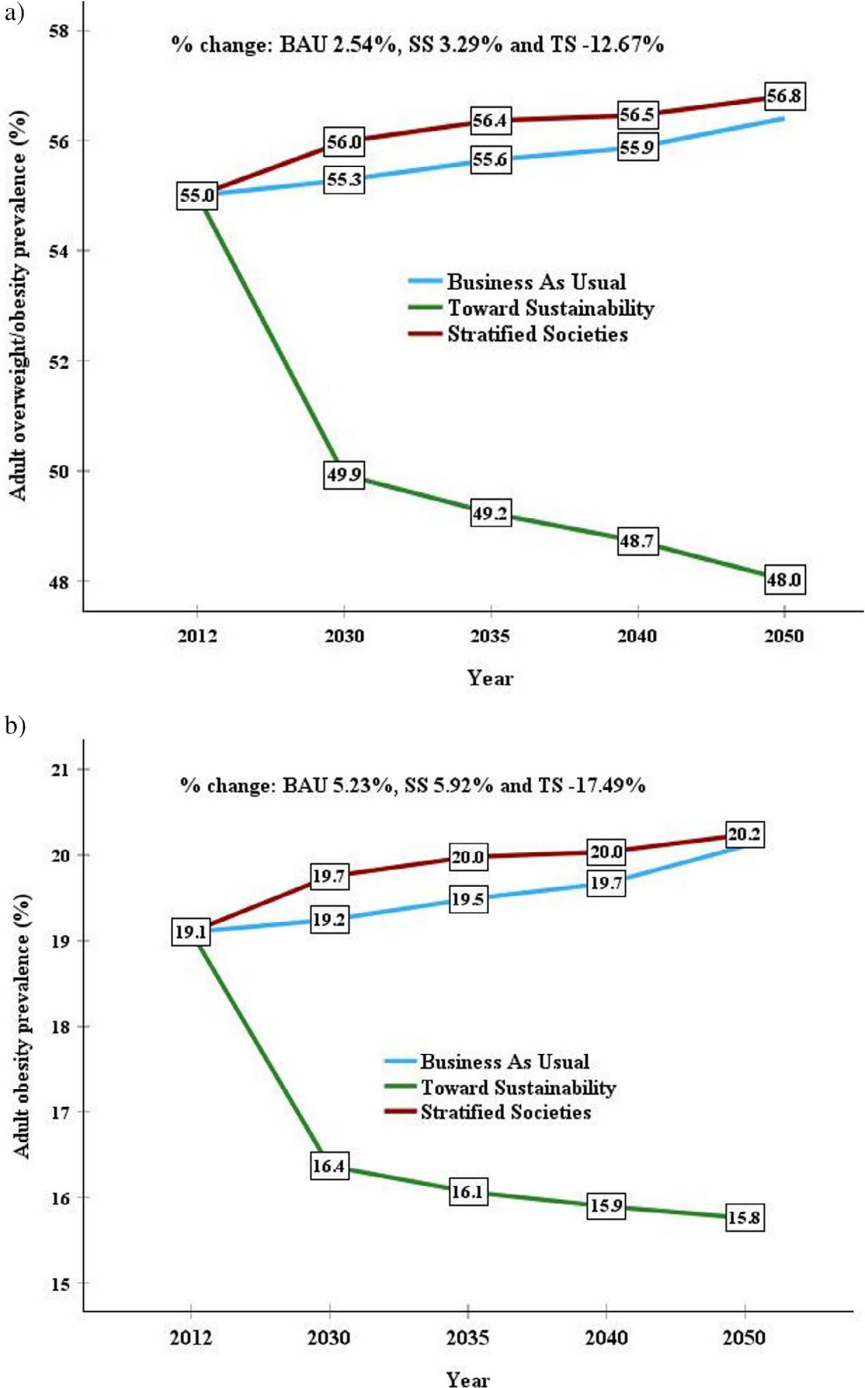
Projections of (a) overweight and obesity (BMI ≥ 25 kg/m^2^) and (b) obesity (BMI ≥ 30 kg/m^2^) prevalences (%) in Spain to 2050 based on three different FAO scenarios (BAU = Business as Usual; SS = Stratified Societies; TS = Toward Sustainability). Data were estimated by a multiple linear regression model based on food nutritive value supplies obtained from FAO food balance sheets from 2000 to 2020 (per capita and day; only food groups containing FAO projections of food supply to 2050). Also presented is the predicted percentage change in prevalences comparing the 2012 BAU with each of the 2050 scenario projections.

Figure 3 shows projections of overweight and obesity (BMI ≥ 25 kg/m^2^) and obesity (BMI ≥ 30 kg/m^2^) prevalences (%) in Spain toward 2050, based on the three FAO scenarios (BAU, SS, and TS). It also shows the predicted proportion of change in prevalence comparing the 2012 BAU with each of the 2050 scenario projections. From a whole perspective, a slight increase (≤ 5%) in overweight and obesity was predicted to 2050 across BAU and SS scenarios when compared to the BAU 2012 baseline, whereas a moderate decrease (≤ 18%) was predicted in the TS scenario. From 2012 to 2050 and using projection data, the proportion of change in overweight and obesity was respectively 2.54%, 3.29%, and 12.67% in the BAU, SS, and TS scenarios, whereas the proportion of change in obesity was 5.23%, 5.92%, and −17.49%, respectively.

## 4 Discussion

### 4.1 Practical Utility of Prediction Models

The FAO FBS data (per capita food supplies) has been used to analyze the relationships between food groups and macronutrient intake, as well as prevalence rates of obesity and overweight at the country level (You and Henneberg 2016). To our knowledge, no predictive models of health outcome variables (adult overweight and obesity), based on that international FAO database, have been reported. Although many of the food nutritive value supplies and signs of the regression coefficients retained in the prediction models are expected results, from a purely statistical viewpoint, the regression model does not necessarily connote causality in the relationship between variables. When interpreting a regression coefficient associated with one of the regressors, we keep the rest of the explanatory variables constant. Therefore, the sign of the partial regression coefficient of a variable may not be the same as that of the simple correlation coefficient between that variable and the dependent one. This is due to the adjustments carried out to obtain the best possible prediction equation.

Energy intake is considered a major influential factor in increasing obesity rates. Food components such as sugar and cereals in diets have been associated with contributing to overweight and obesity in adults (Roccisano and Henneberg 2012; Green et al. 2016). On the other hand, according to You and Henneberg (2016), although different investigations have found that increased intake of dietary fat increases obesity development, increases in obesity cannot be attributed only to changes in dietary fat due to the difficulty in establishing a causal relationship between fat consumption and obesity prevalence. Additionally, increasing studies have noted a greater impact of some dietary patterns on body weight gain than others (Mozaffarian et al. 2011; You and Henneberg 2016). In terms of food groups, the consumption of large quantities of meat has been associated with increased weight gain due to its high energy density and fat content (Schulz et al. 2002; Bes‐Rastrollo et al. 2006; Vergnaud et al. 2010). Our results align with those of You and Henneberg (2016), who found a strong association between meat protein availability and the prevalence of obesity and overweight. Specifically, in that study meat availability received the most significant predictors in the stepwise MLR analysis. However, You and Henneberg (2016) also found that animal protein (excluding meat protein) was associated with those prevalences, but not as significantly as meat protein, which can be explained since other sources of animal protein, such as dairy and fish products, do not contribute to body weight gain (Belobrajdic et al. 2004; Ramel et al. 2009).

From an analytical perspective, the RPD must be > 2 for a predictive model to be useful (Williams 2007). Although the present study was not a laboratory investigation, the obtained prediction models had an RPD in validation close to or greater than this threshold. Regarding the CCC parameter, a value between 0.21 and 0.40 shows fair predictive ability, between 0.41 and 0.60 moderate predictive ability, between 0.61 and 0.80 substantial predictive ability, and between 0.81 and 1.00 accurate predictive ability (Lawrence and Lin 1989; Visentin et al. 2015). The obtained prediction models of the health outcome variables (adult overweight and obesity) showed an accurate predictive ability (CCC > 0.80, Table 2). Considering the bias parameter, no prediction model in the present study significantly overestimated or underestimated the reference values for any of the analyzed variables. Regarding the MLR models used for the projections of adult overweight and obesity prevalences, although the RPD was < 2, the prediction models obtained also showed accurate predictive ability (CCC ≥ 0.80; Table S1).

### 4.2 Trends in Animal‐ and Vegetable‐Sourced Foods in Spain and Long‐Term Future Projections

The use of FBS data to understand food consumption in a country has some limitations, usually in terms of overestimated values, due to the existence of aspects not considered (e.g., processed foods, household waste, nutrient compositions). Additionally, the FAO obtains the FBSs from data provided by the member countries, so FBS reliability depends on the completeness and accuracy of the submitted reports (Thar et al. 2020). Nevertheless, FBS data show an overall pattern of the food supply of countries and provide useful information to build historical trends (Thar et al. 2020). To compare trends using FBS and consumption surveys, managing FBS data in relative terms (i.e., percentages or ratios) is recommended (Rodríguez‐Artalejo et al. 1996; Sheehy and Sharma 2011).

To analyze food supply trends in Spain, data in the present study are reported as both absolute and relative (%) values and further compared with recent studies also carried out in Spain, developed based on institutional food consumption surveys (Varela‐Moreiras et al. 2013), cross‐sectional nutrition studies (ANIBES study; Ruiz et al. 2015, Partearroyo et al. 2019), or a new methodological approach correcting official sources of Spanish food consumption (Cerrillo et al. 2023). As expected, the results obtained were generally higher than those presented in the aforementioned studies. However, some food groups do not meet the current dietary recommendations for the Spanish population, specifically those of the Spanish Society of Community Nutrition (SENC; Aranceta‐Bartrina et al. 2019). The evolution of whole consumption of food groups in Spain has experienced small or moderate variations since 2000; although some trends can be considered negative, others could be positive. Nevertheless, several studies reflect that over the last decades, the adherence to the traditional and healthy Mediterranean diet of the Spanish population has decreased (Partearroyo et al. 2019; Cerrillo et al. 2023).

Milk and dairy products are among the most supplied in the Spanish diet (415 and 429 g/person/day in 2015 and 2020, respectively, Table 3). Although the absolute values were higher than those reported by previous works (Varela‐Moreiras et al. (2013): 359 g/day in 2012; Partearroyo et al. (2019): 257 g/day in 2013; Cerrillo et al. (2023): 215 g/day in 2017), consumption has consistently decreased over time, whether slight as in the present study or significant according to results from Varela‐Moreiras et al. (2013). Despite this higher milk and dairy product consumption, it remains under recommendations, according to Cerrillo et al. (2023), with a ratio of consumed to recommended servings (Rcr) = 0.8. The second most consumed group in the present study was cereals and derivatives, with bread as the main food (Varela‐Moreiras et al. 2013; Ruiz et al. 2015). The current value (313 g/person/day in 2015 and 2020) is consistent with that shown by Cerrillo et al. (2023) for 2017 (350 g/day) but higher than that of Varela‐Moreiras et al. (2013) for 2012 (218 g/day) and Partearroyo et al. (2019) for 2013 (149 g/day). Our results showed a moderate increase (15%) over the last 20 years (Table 3). In contrast, Varela‐Moreiras et al. (2013) highlighted a small variation from 2000 to 2012 but a notable decrease over the last 40 years. In any case, and according to Cerrillo et al. (2023), the current consumption of this food group would cover the dietary recommendations (Rcr = 1).

The amount of vegetables and starchy roots (292 and 160 g/day in 2020, respectively) was greater than the vegetable and green values, including potatoes, as reported by other studies (Varela‐Moreiras et al. 2013; Partearroyo et al. 2019; Cerrillo et al. 2023). On the other hand, the consumption of fruit in 2020 (269 g/day) was similar to the values of around 300 g/day reported by Varela‐Moreiras et al. (2013) and Cerrillo et al. (2023) and higher than the 158 g/day reported by Partearroyo et al. (2019). Regarding trends, FBS data reflected a significant reduction from 2000 to 2020 in vegetables and starchy roots (35% and 24%, respectively) in Spain, with a slight decrease in fruits (Table 3). Varela‐Moreiras et al. (2013) reported a slight increase in these food groups, except for a more pronounced decrease in the consumption of potatoes, from 2000 to 2012. Recommendations for the Spanish population (at least 300 g/day of vegetables, two servings; up to 400 g/day of fruits, three servings) concerning these relevant foods of the Mediterranean diet are still not being met (Rcr of 0.7 and 0.5 for vegetables and fruits, respectively; Cerrillo et al. 2023).

Although our results in absolute values of the meat and derivatives group (279 and 289 g/day in 2015 and 2020, respectively) were higher than those presented by previous works (Varela‐Moreiras et al. (2013): 181 g/day in year 2012; Partearroyo et al. (2019): 146 g/day in year 2013; Cerrillo et al. (2023): 200 g/day in year 2017), similar trends were noted when comparing with Varela‐Moreiras et al. (2013) results, observing a moderate decrease in this food group consumption in recent years. Nevertheless, the mean consumption of meat, particularly red meat, remains higher compared to the dietary recommendation of a maximum of three servings (125 g each)/week (Rcr = 3.6, Cerrillo et al. 2023). Other notable protein sources in the Mediterranean diet are fish and shellfish, with an average consumption slightly higher (119 and 112 g/day in 2015 and 2020, respectively) than those reported by Varela‐Moreiras et al. (2013), Partearroyo et al. (2019), and Cerrillo et al. (2023), with 89 g/day in 2012, 63 g/day in 2013, and 74 g/day in 2017, respectively. In concordance with our data, Partearroyo et al. (2019) indicated that their consumption decreased slightly in the past few years. However, consumption can be considered beneficial and adjusted to the recommendations (Rcr = 1, Cerrillo et al. 2023).

Other minor foods included in the Mediterranean diet are pulses and legumes, eggs, nuts, and vegetable oils, mainly olive oil. Oils and fats constitute the most important sources of lipids for the Spanish population; 90% of the total intake is of vegetable origin, notably olive oil (Varela‐Moreiras et al. 2013; Partearroyo et al. 2019). The absolute values of oils and fats from our study (77 and 13 g/day in 2015; 84 and 13 g/day in 2020, respectively) are higher than those of total oils and fats shown by previous works (Varela‐Moreiras et al. (2013): 42 g/day in 2012; Partearroyo et al. (2019): 25 g/day in 2013; Cerrillo et al. (2023): 34 g/day in 2017). Regarding the consumption trend, although Varela‐Moreiras et al. (2013) and Partearroyo et al. (2019) indicated a considerable global reduction for this food group between 2000 and 2013, our study reveals a slight increase in vegetable oils and a slight reduction in animal fats from 2000 to 2020. According to Cerrillo et al. (2023), the current consumption of this food group would still not cover the dietary recommendations (Rcr = 0.7).

The results on egg consumption (38 and 41 g/day in year 2015 and 2020, respectively) were similarly higher than those reported by Varela‐Moreiras et al. (2013), Partearroyo et al. (2019), and Cerrillo et al. (2023), at 27 g/day in year 2012, 29 g/day in year 2013, and 34 g/day in year 2017, respectively. Regarding the consumption trend, whereas Varela‐Moreiras et al. (2013) and Partearroyo et al. (2019) indicated a slight reduction from 2000 to 2013, our data show a slight increase from 2000 to 2020. According to the SENC (Aranceta‐Bartrina et al. 2019), the intake of three to five egg units/week would represent a valid nutritional alternative to meat and fish products, recommendations that would be met in the current average diet (Rcr = 1, Cerrillo et al. 2023).

Concerning legumes and pulses, the supply data of our study (14 and 16 g/day in 2015 and 2020, respectively) are similar to those indicated in previous studies (Varela‐Moreiras et al. 2013; Partearroyo et al. 2019; Cerrillo et al. 2023), at 13 g/day in 2012, 14 g/day in 2013, and 15 g/day in 2017, respectively. In agreement with our data, Varela‐Moreiras et al. (2013) indicated that consumption had increased slightly in the past few years. One objective of the SENC dietary guidelines (Aranceta‐Bartrina et al. 2019) is that the Spanish population consumes approximately 60 to 80 g of pulses at least two to four times/week, but currently only half of this recommendation is consumed (Rcr = 0.5, Cerrillo et al. 2023).

Regarding nuts, the consumption in our study (15 and 36 g/day in 2015 and 2020, respectively) was higher than the 2.6 g/day in 2017 reported by Cerrillo et al. (2023). Although this consumption has remained steadily low for most of the last 20 years (Table 3), it is seemingly experiencing a current increase. Nuts are another group of foods that the SENC (Aranceta‐Bartrina et al. 2019) recommends increasing consumption in the Spanish population, specifically approximately 25 g servings at least three to seven times/week. However, according to Cerrillo et al. (2023), current consumption is far from these recommendations (Rcr = 0.14).

Another minor food group not included in the Mediterranean diet, but a component in the current diet that has been associated with overweight and obesity in adults, is sugars and sweeteners. Their supply according to the FBS data (91 and 85 g/day in 2015 and 2020, respectively; Table 4) is similar to the results obtained by Cerrillo et al. (2023; 76 g/day in 2017) but higher than those reported by Varela‐Moreiras et al. (2013; 26 g/day in 2012) and Partearroyo et al. (2019; 16 g/day in 2013). The trend over time has not been discussed in other recent studies, but according to our data, the consumption of sugars and sweeteners has increased slightly in the past few years.

The mean energy supply per capita for the Spanish population according to FBS data (3195 and 3354 kcal/day in 2015 and 2020, respectively; Table 4) is similar to the results obtained by Cerrillo et al. (2023; 2930 kcal/day in 2017) but higher than those reported in other recent studies (Varela‐Moreiras et al. 2013; Ruiz et al. 2015; 2609 kcal/day in 2012 and 1810 kcal/day in 2013, respectively). However, our data, similar to those reported by Varela‐Moreiras et al. (2013) on dietary caloric consumption, reveal a slight decrease over the last few years. Likewise, the mean protein intake per capita for the Spanish population according to the FBS data (105 and 111 g/day in 2015 and 2020, respectively; Table 4) is similar to the values shown in previous studies (Varela‐Moreiras et al. 2013; Cerrillo et al. 2023; 94 g/day in 2012 and 107 g/day in 2017, respectively). The trends for this macronutrient intake from 2000 to the present also indicate a slight decrease (Table 4; Varela‐Moreiras et al. 2013). According to the European Food Safety Authority (2017), the daily consumption of these macronutrients is above the current recommended daily intake (RDI) for both energy (1906–2156 kcal/day) and protein (52–76 g/day).

The proportional contribution of carbohydrates to total energy intake (45% in both 2015 and 2020; Table 4) is slightly higher than those obtained in other Spanish studies (Varela‐Moreiras et al. 2013; Ruiz et al. 2015; Cerrillo et al. 2023; 42% in 2012, 41% in 2013, and 42% in 2017, respectively) and at the lower limit of recommendations (RDI: 45%–60%). The proportion of lipids in our study presents similar values (42% in both 2015 and 2020) to those of other national studies (Varela‐Moreiras et al. 2013; Ruiz et al. 2015; Cerrillo et al. 2023; 43%, 39%, and 40%, respectively). In general, these values exceeded the recommendations (RDI: 20%–40%). Moreover, evidence indicates that to avoid unhealthy weight gain, total fat should not exceed 30% of total energy consumption (WHO 2018). Carbohydrates, which historically in the Spanish diet have been linked to the consumption of cereals, pulses, and potato groups (Varela‐Moreiras et al. 2013), could have been replaced by energy intake from lipids of ultra‐processed foods (Varela‐Moreiras et al. 2013; Cerrillo et al. 2023), characterized by large amounts of saturated fats and free sugars (del Martí Moral et al. 2021). In contrast, the proportional contribution of protein in our study (13% in both 2015 and 2020) was slightly lower than those indicated in previous studies (Varela‐Moreiras et al. 2013; Ruiz et al. 2015; Cerrillo et al. 2023; 15%, 17%, and 15%, respectively) and at or slightly below the highest limit of recommendations (RDI: 10%–15%). Regarding trends, the proportions of carbohydrates and lipids have practically not changed since 2000 (Table 4; Varela‐Moreiras et al. 2013), but the proportion of protein has decreased slightly due to the decrease in the proportion of energy from animal products (Table 4).

Concerns are increasing about the sustainability of food production from the perspective of both planetary and human health (FAO 2018a), particularly regarding animal‐based foods (Henchion et al. 2021). The outcomes of the BAU and SS scenarios regarding consumption to 2050 (Table 5) show that per capita demand will increase over time. However, the TS scenario predicts a decrease in consumption and would be more in line with the current food‐based dietary guidelines (FBDGs) of some countries such as Spain, which promote a more sustainable and healthy diet (IPCC 2019; Aranceta‐Bartrina et al. 2019). In those FBDGs that include sustainability measures, for example, recommendations exist to prioritize plant‐based foods and limit red and processed meat (Henchion et al. 2021). However, none of the FAO scenarios would completely cover the expectations of daily per capita recommendations for the Spanish population analyzed previously. Although a decrease in total energy and protein, sugars, and meat is desirable, the consumption of vegetables, starchy roots, and fruits should be increased. Additionally, all scenarios projected a significant decrease in fish consumption, despite its being a relevant food, mainly due to its high content of omega‐3 fatty acids (Rimm et al. 2018).

### 4.3 Trends of Overweight and Obesity in Spain and Long‐Term Future Projections

According to the data of the present study and considering obesity and overweight together, more than half (55% in 2020) of the Spanish population older than 18 years are overweight, and around 20% are obese (Figure 2). The values of overweight estimated by regression in this study are comparable with the estimates obtained in the ANIBES study (López‐Sobaler et al. 2016) for 2013 (35%) and the latest Spanish Health Survey (ENSA) data (37%) in 2017 (MSCB 2017). The values from the ENPE study (Aranceta‐Bartrina et al. 2016) from 2014 and 2015 and the EHSS from 2009 and 2014 (Acevedo et al. 2017) were slightly higher (approximately 40%). In the ANIBES and ENPE studies, which are based on anthropometric data, the prevalence of obesity (21% and 20%, respectively) was consistent with the estimates obtained in the present study. It was somewhat higher than that observed in studies estimating the prevalence of overweight and obesity from self‐reported data (EHSS and ENSA surveys), which could be explained because people with obesity tend to under‐report their body weight (Nyholm et al. 2007; Acevedo et al. 2014). The high caloric intake beyond the population's needs would partly explain Spain's high overweight and obesity rates in adults (Cerrillo et al. 2023).

Regarding time trends, the main purpose of this study, NCD‐RisC data between 2000 and 2016 (Figure 2) showed an upward trend in overweight and obesity prevalences in the adult Spanish population. The latest ENSA data (MSCB, 2017) also highlight this trend, indicating that in the last 30 years, the prevalence of obesity in adults has multiplied by 2.4, from 7.4% in 1987 to 17.4% in 2017. This trend also aligns with the projections of overweight and obesity prevalences in Spain to 2050 based on the BAU and SS scenarios when compared to the BAU 2012 baseline since these scenarios predict that the consumption per capita (especially the total energy and protein) will increase.

The data obtained by MLR models in this study showed a trend toward lower overweight and obesity prevalence in the last decade, whereas the prevalence of obesity remained virtually the same (Figure 2). This positive trend is consistent with that detected in the single 5‐year period (from 2009 to 2014) of the EHSS study (Acevedo et al. 2017), despite the limitation of analyzing self‐reported data. This trend also aligns with the projections of prevalence in Spain to 2050 according to the TS scenario, which predicts a decrease in consumption per capita, and would be more in line with the current FBDGs (more sustainable and healthy diet) of Spain (IPCC 2019; Aranceta‐Bartrina et al. 2019). It will be necessary to assess whether this trend remains stable and whether it is the result of changes in dietary patterns and lifestyles, promoted by sectors involved in public health.

## 5 Conclusions

The accuracy of MLR models to predict adult overweight and obesity prevalences in each country using FAO FBS data (annual changes in the daily per capita food supplies) was high, as was their predicting ability. The FBSs are updated regularly and standardized for all countries. Therefore, and using within‐country analysis, the models obtained could help to monitor these health outcome variables and examine the effects of policies and interventions.

The evolution of whole consumption of food groups in Spain has undergone small or moderate variations since 2000; some trends can be considered negative, whereas others are positive. However, this work, along with other published studies, reflects that over recent decades, the adherence to the traditional and healthy Mediterranean diet of the Spanish population has decreased.

The overweight and obesity prevalences in Spain estimated by MLR showed a trend toward lower levels or remained virtually the same over the last decade. This trend aligns with the projections of prevalence in Spain to 2050 according to the TS scenario, which predicts a decrease in consumption per capita and would be more in line with the current FBDGs (more sustainable and healthy diet) of Spain. It will be necessary to assess whether this trend remains stable and whether it is the result of changes in dietary patterns and lifestyles, promoted by sectors involved in public health.

## Author Contributions

**Manuel Delgado‐Pertíñez:** conceptualization, methodology, data curation, formal analysis, resources, investigation, validation, writing original draft; **Sara Muñoz‐Vallés:** data curation, visualization, writing – original draft; writing – review and editing; **José Luis Guzmán:** data curation, formal analysis, investigation, writing – review and editing; **Luis Ángel Zarazaga:** visualization, formal analysis, investigation, writing – review and editing; **Mao Chou Hsu:** conceptualization, methodology, data curation, formal analysis, resources, investigation, validation, writing original draft; **Michael López‐Herrera:** conceptualization, visualization, resources, writing – original draft, supervision, writing – review and editing.

## Funding

Open Access funding provided thanks to the CRUE‐CSIC agreement.

## Consent

Informed consent was obtained from all subjects involved in the study.

## Conflicts of Interest

The authors declare no conflicts of interest.

## Supporting information


**Table S1:** Final multiple linear regression model based on food nutritive value supplies obtained from the FAO's food balance sheets from 2000 to 2020 (per capita and day; only food groups that contains FAO projections of foods supply to 2050) and validation for the prediction of adult overweight and obesity (BMI ≥ 25 kg/m^2^) and obesity (BMI ≥ 30 kg/m^2^) prevalences on three different FAO scenarios.
**Figure S1:** Graphic representation of the calculated values of (a) adult overweight and obesity (BMI ≥ 25 kg/m^2^) and (b) obesity (BMI ≥ 25 kg/m^2^) prevalences and their estimation from multiple linear regression with split sample validation (30%) using the developed prediction model from Table S1.

## Data Availability

The data presented in this study are available on request from the corresponding author.
