# Supplementary material for: Predicting Adult Overweight and Obesity Prevalences Using the Food Nutritive Value Supplies of the FAO's Food Balance Sheet Data: Case Study of Trends in Spain
Source: Food Sci Nutr. 2026 Feb 22;14(2):e71567. doi: 10.1002/fsn3.71567 (PMC12928066; doi:10.1002/fsn3.71567)

**Predicting adult overweight and obesity prevalences using the food nutritive value supplies of the FAO’s food balance sheet data: Case study of trends in Spain**

**Table S1**

Final multiple linear regression model based on food nutritive value supplies obtained from the FAO’s food balance sheets from 2000 to 2020 (per capita and day; only food groups that contains FAO projections of foods supply to 2050) and validation for the prediction of adult overweight and obesity (BMI ≥ 25 kg/m^2^) and obesity (BMI ≥ 30 kg/m^2^) prevalences on three different FAO scenarios.

| Item^1^ | Adult overweight and obesity prevalence (%) | | | | | |  | Adult obesity prevalence (%) | | | | | |
| --- | --- | --- | --- | --- | --- | --- | --- | --- | --- | --- | --- | --- | --- |
|  | n | R^2^ | SEP^2^ | Regression coefficient | SE | P-value |  | n | R^2^ | SEP^2^ | Regression coefficient | SE | P-value |
| Calibration model | 1848 | 0.73 | 8.29 |  |  |  |  | 1848 | 0.68 | 4.59 |  |  |  |
| Intercept |  |  |  | 12.86 | 1.142 | <0.001 |  |  |  |  | -2.283 | 0.619 | <0.001 |
| E sugars |  |  |  | 0.041 | 0.002 | <0.001 |  |  |  |  | 0.024 | 0.001 | <0.001 |
| P cereals |  |  |  | 1.178 | 0.058 | <0.001 |  |  |  |  | 0.492 | 0.033 | <0.001 |
| E cereals |  |  |  | -0.023 | 0.002 | <0.001 |  |  |  |  | -0.008 | 0.001 | <0.001 |
| P oil crops |  |  |  | -1.076 | 0.104 | <0.001 |  |  |  |  |  |  |  |
| E fruits |  |  |  | 0.029 | 0.003 | <0.001 |  |  |  |  | 0.015 | 0.001 | <0.001 |
| P meat |  |  |  | 0.547 | 0.026 | <0.001 |  |  |  |  | 0.287 | 0.015 | <0.001 |
| E fish and seafood |  |  |  |  |  |  |  |  |  |  | -0.029 | 0.003 | <0.001 |
| Population, million |  |  |  |  |  |  |  |  |  |  | -0.006 | 0.001 | <0.001 |
| Validation | 823 | 0.69 | 8.54 |  |  |  |  | 829 | 0.66 | 4.60 |  |  |  |

^1^ E, energy (kcal); P, protein (g). ^2^ SEP_,_ standard error of prediction.

**Figure S1**

Graphic representation of the calculated values of (a) adult overweight and obesity (BMI ≥ 25 kg/m^2^) and (b) obesity (BMI ≥ 25 kg/m^2^) prevalences and their estimation from multiple linear regression with split sample validation (30%) using the developed prediction model from Table S1.

a)


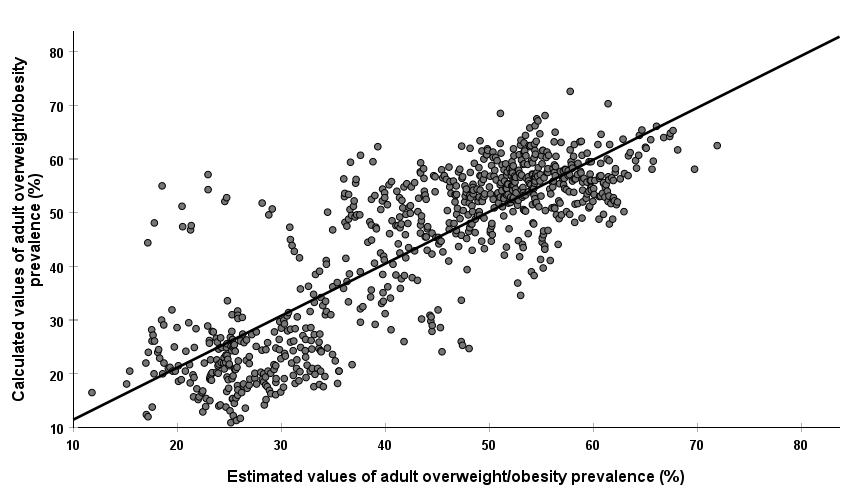


b)


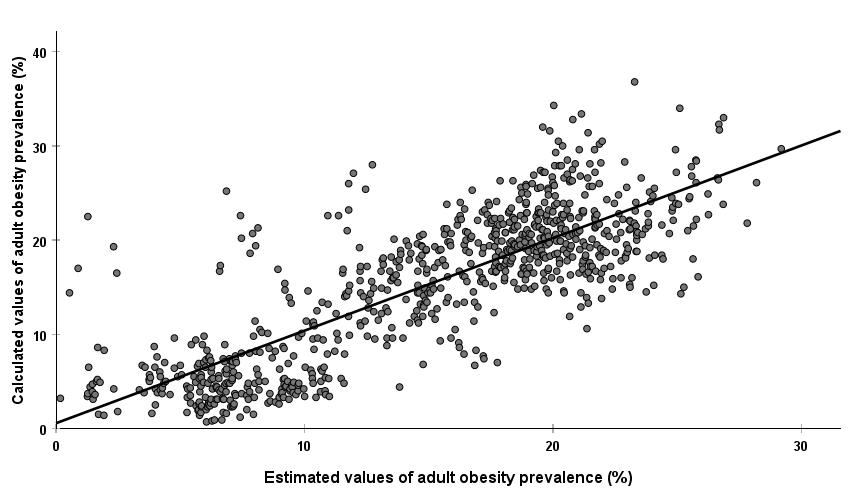

Supplement: Supplementary file 1 — Table S1: Final multiple linear regression model based on food nutritive value supplies obtained from the FAO's food balance sheets from 2000 to 2020 (per capita and day; only food groups that contains FAO projections of foods supply to 2050) and validation for the prediction of adult overweight and obesity (BMI ≥ 25 kg/m2) and obesity (BMI ≥ 30 kg/m2) prevalences on three different FAO scenarios. Figure S1: Graphic representation of the calculated values of (a) adult overweight and obesity (BMI ≥ 25 kg/m2) and (b) obesity (BMI ≥ 25 kg/m2) prevalences and their estimation from multiple linear regression with split sample validation (30%) using the developed prediction model from Table S1. [file FSN3-14-e71567-s001.docx]
